# Doctors' Wills

**Published:** 1914-05-02

**Authors:** 


### Doctors' Wills.

Dr. George Moore, M.D., of Shirehampton, Bristol, Deputy-Inspector-General of Hospitals and Fleets, R.N. (retired), who saw service during the Russian War, 1854-5, and in Vancouver Island in 1856, has left estate valued at £1,857.

Dr. William Watts Thetford, M.D., L.R.C.P., M.R.C.S., of Strangford, County Down, surgeon to the Coastguard and medical officer to the Royal Irish Constabulary, has left personal estate in the United Kingdom valued at £36,603 8s. 1d. He left £100 to the Royal Medical Benevolent Fund Society of Ireland.

